# The Mediating Role of Spirituality in the Effect of the Nursing Work Environment on the Quality of Working Life: A Structural Equation Modeling Study

**DOI:** 10.1155/jonm/5357100

**Published:** 2025-10-22

**Authors:** Sevgi Koroglu

**Affiliations:** ^1^Department of Nursing, Faculty of Health Sciences, Sakarya University, Sakarya, Türkiye; ^2^Institute of Health Sciences, Sakarya University, Sakarya, Türkiye

**Keywords:** nurses, occupational health, spirituality, workplace

## Abstract

**Background:**

Nurses are working in increasingly complex environments due to technological advances and expanding role expectations. These environments lead to issues such as burnout and job dissatisfaction, which negatively affect the quality of working life. Although the relationship between the work environment and quality of working life has been explored in the literature, the mediating role of spirituality in this relationship remains insufficiently studied.

**Objective:**

The aim of this study is to examine the relationship between nurses' perceptions of their work environments and their quality of working life and to test whether spirituality is a mediating factor in this effect.

**Methods:**

This descriptive and cross-sectional study was conducted online with 350 nurses working across Türkiye between June and July 2025. Data were collected using the Nursing Job Index, Nursing Work Environment Assessment Scale, the Employee Spirituality Scale, and the Nursing Work–Life Quality Scale. The data were analyzed using structural equation modeling (SEM). SPSS Version 25.0 and R software Version 4.4.2 were used for the analyses.

**Results:**

The findings indicate that work environments perceived positively by nurses are associated with higher levels of spirituality and better work–life quality. Spirituality was found to serve as a mediating factor in the relationship between the work environment and the quality of working life.

**Conclusions:**

A positive nursing work environment is directly linked to a higher quality of working life through spirituality. The existence of environments sensitive to spiritual needs appears to be linked to nurses' inner resources, and this may be related to professional well-being and service quality. Further qualitative research in diverse cultural contexts is needed to gain a deeper understanding of the role of spirituality in the quality of working life.

## 1. Introduction

Healthcare services are rapidly evolving due to technological developments, demographic changes, and increasing patient needs [1]. Nurses, who represent a key component of the healthcare workforce, have gradually moved beyond their traditional caregiving roles. Today, they play active roles in management, leadership, policymaking, and digital health practices [2]. With advanced education, nurses have begun to take on positions that allow them to influence organizational culture and service delivery, making them essential coordinators and communicators in modern healthcare systems [3, 4]. However, this expansion in roles and responsibilities has also brought significant challenges. Nurses face increased patient loads, staff shortages, extended working hours, multiple responsibilities, digital system adaptation, and role confusion [5–8]. These issues have been linked to burnout, compassion fatigue, and job satisfaction, all of which can negatively impact nurses' health and the quality of care provided [9, 10].

Creating positive clinical work environments is essential to help nurses cope with these challenges. Effective communication, authentic leadership, meaningful recognition, adequate staffing, and organizational support are factors associated with a healthy working environment; they are also linked to nurses' satisfaction and quality of care, as indicated in the literature [11, 12]. In addition, comprehensive improvement strategies that address the task-related, social, physical, and cultural dimensions of the work environment strengthen job satisfaction and professional autonomy [13]. Conversely, working environments characterized by weak management support, ineffective communication, heavy workloads, ethical conflicts, and poor organizational culture have been reported to be negatively associated with nurses' well-being and the quality of care [14–16].

Nurses' working environments significantly shape their quality of working life. Various studies conducted in different societies have demonstrated that nurses generally report a moderate level of professional quality of life [17–19]. Supportive and functional working environments are reported to be associated with higher occupational quality of life, and occupational quality of life is positively linked to job satisfaction [20]. Factors such as shorter and more balanced working hours, psychological support, harmonious teamwork, social security, clearly defined roles, and effective management support are also positively associated with nurses' quality of professional life [21]. Furthermore, favorable nursing practice environments, high levels of empathy, strong social support, and compassion satisfaction are reported to be associated with a higher quality of life [22].

The job demands–resources (JD-R) model is a comprehensive theoretical framework used to explain employees' occupational quality of life. The model posits that every job is built upon two fundamental dimensions: job demands, which consume employees' physical and mental energy (e.g., heavy workload, long shifts, role conflicts, and emotionally demanding caregiving responsibilities), and job resources, which mitigate the negative effects of these demands while also enhancing motivation and job satisfaction (e.g., supportive leadership, open communication, adequate staffing, team cohesion, and constructive feedback) [23]. A key feature of the JD-R model is that it focuses not only on environmental factors but also on employees' personal resources [24]. Personal resources reflect individuals' internal strengths, such as self-efficacy, resilience, hope, and positive self-perception; these elements both provide resistance to the demanding aspects of job demands and amplify the benefits of existing job resources [23, 24]. At this point, spirituality, associated in the literature with alleviating anxiety and depression [25], facilitating coping with stress [26, 27], and posttraumatic growth [27], is considered a powerful personal resource within the JD-R framework [23, 24]. Spirituality is defined as the process of discovering and expressing one's connection to the self, others, nature, the meaningful, or the sacred. It differs from religiosity and is instead linked to an individual's sense of meaning, purpose, and inner strength [28]. Religiosity denotes the commitment to the doctrines, rituals, and regulations of a specific religion, whereas spirituality encompasses an individual's introspective quest for significance and profound connection with existence. Religiosity encompasses external and visible actions, including rituals and worship, whereas spirituality pertains to an internal experience that may exist outside of religious frameworks. In this regard, spirituality is a more expansive and subjective notion than religiosity [29]. The inclusive and transreligious essence of spirituality is evident in both personal and professional domains. While the literature largely focuses on nurses' roles in delivering spiritual care [30, 31], the influence of nurses' own spirituality on their professional lives has received limited attention. Nevertheless, research indicates that nurses with higher levels of spirituality tend to report greater job satisfaction, better emotional stability, and stronger mental well-being [32, 33]. These nurses are also more competent in providing holistic and patient-centered care, which has been associated with positive patient outcomes [34]. In addition to individual spirituality, the presence of a supportive spiritual climate at the organizational level is also crucial. A spiritually supportive environment has been correlated with job satisfaction and a more meaningful professional experience [35, 36]. Moreover, fostering spirituality in the workplace has been associated with improvements in leadership style, organizational commitment, and employee motivation. Spiritual leadership, a sense of meaning, and spiritual support have been found to be associated with nurses' productivity and organizational commitment, offering healthcare organizations a strategic advantage [37].

Although previous research has explored the relationship between the nursing work environment and the quality of working life, spirituality as a mediating factor in this relationship has not been sufficiently examined. Therefore, the primary objective of this study is to evaluate the relationship between nurses' working environment and their quality of working life, as well as to test the potential mediating role of spirituality in this relationship. The study seeks to discover organizational elements potentially associated with nurses' professional well-being. The research was conducted in accordance with the following hypotheses:

• H1: A more positive perception of the work environment among nurses is associated with higher levels of spirituality.
• H2: Higher levels of spirituality among nurses are positively associated with their quality of working life.
• H3: Spirituality plays a potential mediating role in the relationship between nurses' perception of their work environment and their quality of working life.

## 2. Methods

### 2.1. Setting and Sample

This descriptive, cross-sectional study was conducted online in Türkiye between June and July 2025 using purposive sampling. Participants were recruited through various online platforms, including WhatsApp, Instagram, and X. Nurses were deemed eligible to participate if they met the following inclusion criteria: (1) working in a healthcare facility that provides inpatient care, (2) having at least 1 year of professional experience, (3) serving as the primary caregiver for a patient, and (4) voluntarily agreeing to participate in the study.

Exclusion criteria were as follows: (1) employment in outpatient care settings such as family health centers, healthy life centers, community mental health centers, or hospital outpatient departments; (2) inability to actively practice nursing during the study period for any reason; and (3) employment in administrative or managerial positions. Nurses in managerial roles were excluded due to the significant differences in their job descriptions, patient interaction levels, and workloads compared to clinical nurses. Similarly, outpatient care settings were excluded because of their limited and less direct patient interaction. These two groups were excluded to preserve the validity and reliability of the study findings by ensuring a consistent participant profile centered on direct patient care.

The required sample size was calculated using G∗Power 3.1 software, based on the correlation coefficient (*r* = −0.152) reported in a prior study by Karaman and Altınel, which investigated the relationship between perceived stress and quality of working life among nurses [38]. Using this correlation coefficient, a power analysis was conducted with an assumed effect size of *r* = 0.15, a two-tailed test, a significance level of *α* = 0.05, and a statistical power of 0.80. The analysis indicated that a minimum of 346 participants would be required to achieve sufficient power for the study.

### 2.2. Recruitment and Data Collection

An announcement outlining the purpose and significance of the study, along with the researcher's contact information, was initially disseminated through various online platforms. Nurses who volunteered to participate after viewing the announcement were provided with detailed information regarding the study's purpose, scope, potential benefits, data confidentiality, the intention to publish the results in national or international journals, and their right to withdraw from the study at any stage during data collection.

Those who remained willing to participate provided informed consent by signing and returning the consent form, which was sent to them via the researcher's email. Upon receipt of the signed consent form, participants were sent a link to the online questionnaire and measurement scales via Google Forms. All items within the survey were mandatory, and each participant was allowed to complete the form only once.

### 2.3. Data Collection Tools

The Personal Information Form, developed by the researchers, collected data on nurses' demographic characteristics (e.g., gender, age, marital status, and income level), professional attributes (e.g., education level, years of professional experience, type of institution and unit of employment, and weekly working hours), and personal attitudes and practices related to spirituality.

The Nursing Job Index, Nursing Work Environment Assessment Scale, originally developed by Lake, was used to assess nurses' perceptions of their work environment. The Turkish adaptation and validation of the scale were conducted by Türkmen et al. This instrument consists of 31 items rated on a 4-point Likert scale (1 = strongly agree to 4 = strongly disagree), and all items are reverse-scored. The scale comprises five subscales, with subscale scores calculated by averaging relevant items. Higher average scores indicate more positive evaluations of the work environment [39]. In the present study, the scale demonstrated high internal consistency with a Cronbach's alpha coefficient of 0.92.

The Employee Spirituality Scale, developed by Milliman et al., was employed to measure individuals' spiritual orientation within the workplace. The Turkish validity and reliability study was carried out by Apak. This scale includes 22 items and two subdimensions, rated on a 5-point Likert scale (1 = strongly disagree to 5 = strongly agree). Higher scores reflect stronger workplace spirituality [40]. In the current study, the Cronbach's alpha coefficient was 0.85, indicating good reliability.

The Nursing Work–Life Quality Scale, developed by Brooks, was used to evaluate nurses' quality of working life. The Turkish version of the scale was validated by Şirin. This 35-item instrument consists of five subdimensions and is rated on a 5-point Likert scale (1 = strongly disagree to 5 = strongly agree), yielding total scores ranging from 35 to 175. Higher scores indicate higher perceived quality of working life [41]. The Cronbach's alpha coefficient for this scale in our study was 0.84, indicating acceptable reliability.

### 2.4. Data Analysis

Data were analyzed using IBM SPSS Statistics for Windows, Version 25.0, and R software, Version 4.4.2. Structural equation modeling (SEM) was conducted in the R environment using the lavaan package. Descriptive statistics were presented as means and standard deviations or medians (minimum–maximum) for continuous variables, and as frequencies and percentages for categorical variables. The assumption of normality was assessed using both graphical methods (e.g., Q–Q plots, histograms) and the Shapiro–Wilk test. SEM was employed to examine the direct and indirect effects of nurses' perceptions of their work environment on their quality of working life. The structural model results were illustrated through a path diagram. Model fit was evaluated using multiple goodness-of-fit indices (GFIs), including the chi-square (*χ*^2^) test, comparative (CFI), Tucker–Lewis index (TLI), GFI, adjusted GFI (AGFI), normed fit index (NFI), root mean square error of approximation (RMSEA), and standardized root mean square residual (SRMR). Furthermore, the bootstrap method with 5000 samples was applied to derive more robust standard errors and confidence intervals for indirect and total effects. All results were reported with corresponding confidence intervals, and a statistical significance level of *p* < 0.05 was adopted.

### 2.5. Ethical Considerations

Ethical approval for this study was obtained from the Sakarya University Social and Human Sciences Ethics Committee (Reference no: E-61923333-050.99-485874). Written informed consent was obtained from all participants through an online platform using an “Informed Consent Form.” All procedures were carried out in accordance with the ethical standards of the institutional and national research committees and with the principles of the 1964 Declaration of Helsinki, as revised in 2000.

## 3. Results

### 3.1. General Characteristics

A total of 350 nurses participated in the study. All 350 participants met the inclusion criteria and completed the study; therefore, no withdrawals or exclusions occurred, and all responses were included in the final analysis. The participants had a mean age of 29.53 years (SD = 5.17), with ages ranging from 22 to 38 years. Among them, 69.1% (*n* = 242) were female, 54.9% (*n* = 192) were single, and 43.2% (*n* = 306) held an undergraduate degree. In terms of income status, 63.1% (*n* = 221) reported that their income was equal to their expenses. More than half of the participants (53.1%, *n* = 186) had between 6 and 10 years of professional experience. The majority worked in public hospitals (68.9%, *n* = 241), while 37.1% (*n* = 116) were employed in internal medicine clinics. Regarding weekly working hours, 42% (*n* = 147) of participants reported working between 41 and 50 h per week.

In relation to spirituality, 66.9% (*n* = 234) of the participants indicated that they considered spirituality to be important, and 49.1% (*n* = 172) stated that they engage in daily spiritual practices. The demographic and professional characteristics of the participants are detailed in Table 1.

### 3.2. Correlation Analysis

The Pearson correlation analysis among nursing work environment, spirituality, and quality of working life is presented in Table 2. A positive correlation was found between the nursing work environment and spirituality (*r* = 0.57, *p* < 0.001), as well as between the nursing work environment and quality of working life (*r* = 0.31, *p* < 0.001). Furthermore, spirituality was also positively correlated with quality of working life (*r* = 0.30, *p* < 0.001) (Table 2).

### 3.3. Validation Results of the Research Model

The chi-square value was *χ*^2^ (51) = 52.78, *p*=0.405, indicating that the result was not statistically significant. This suggests that there is no significant difference between the model and the observed data, demonstrating a good model fit. The *χ*^2^/df ratio was 1.034, indicating an excellent level of fit. Error-based fit indices were RMSEA = 0.010 and SRMR = 0.023, both of which fall below the accepted threshold (< 0.08). The CFI (0.976), TLI (0.980), and NFI (0.963), all exceeded 0.90, supporting strong structural validity of the model. In addition, the GFI (0.971) and AGFI (0.961) values indicated high explanatory power of the structural model (Table 3).

### 3.4. Path Analysis

A path coefficient estimation and significance test were performed using SEM to verify the relationships between variables. The findings showed that the nursing work environment was positively and significantly related to spirituality (*β* = 0.648, *p* < 0.001) and work–life quality (*β* = 0.220, *p*=0.014). Furthermore, spirituality also showed a positive and significant relationship with work–life quality (*β* = 0.216, *p*=0.021) (Table 4).

### 3.5. Effect Analysis

**Figure 1:**
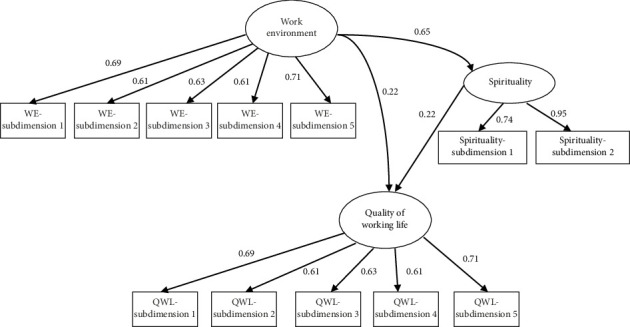
Structural equation modeling of the effect of the nursing work environment on quality of working life through spirituality.

The bootstrap method was used to determine the mediating effect of spirituality in the relationship between the nursing work environment and quality of working life. The analysis results indicate that the nursing work environment is directly and significantly related to the quality of working life (*β* = 0.220, *p*=0.014). In addition, the indirect effect through spirituality was found to be *β* = 0.201, which was also statistically significant (*p*=0.029). The 95% confidence interval for the indirect effect was (0.024, 0.390), and since this interval did not include zero, the presence of a mediating effect is reliably supported. The total effect, which combines both the direct and indirect effects, was calculated as *β* = 0.516 (*p* < 0.001, 95% CI: 0.348, 0.703). Based on these findings, the indirect effect accounts for approximately 39% of the total effect. Therefore, spirituality plays a significant and partial mediating role in the relationship between the nursing work environment and quality of working life (Table 5). The values for total, indirect, and direct effects in the model are presented in Figure 1.

## 4. Discussion

The quality of nurses' working life plays a critical role in ensuring effective patient care, enhancing employee satisfaction, and sustaining the healthcare system. Accordingly, this study aimed to examine the relationship between the nursing work environment and quality of working life, with a particular focus on the mediating role of spirituality. The findings reveal that a positive nursing work environment is significantly associated with work–life quality, both directly and indirectly through spirituality, and that all proposed hypotheses are supported.

These results are consistent with existing literature. Previous research has also demonstrated positive and significant associations between the quality of the nursing work environment and quality of working life [20, 42, 43]. It has been reported that inclusive leadership is associated with job commitment [44], that person–organization fit and workplace happiness are associated with job commitment [45], and that orientation training provided during the pandemic was positively associated with nurses' sense of security and motivation [46]. Collectively, these findings suggest that a supportive and well-structured work environment fosters both the psychological and social well-being of nurses. This holistic influence appears to be a critical determinant in improving the overall quality of working life.

The findings of this study reveal that employee spirituality plays a partial mediating role in the relationship between a positive nursing work environment and quality of working life. This shows that nurses are influenced not only by external conditions but also by internal resources. The literature supports these findings, particularly emphasizing that an individual's levels of meaning, belonging, and faith are related to their quality of professional life. A study using a SEM reported that spirituality was significantly associated with quality of working life; nurses' visions and intrinsic commitment to their work were positively linked to quality of life [47]. Similarly, it has been noted that spiritual awareness and a personalized care approach are negatively associated with compassion fatigue and burnout, and that spirituality plays a moderating role in the relationship between stress and quality of life [48, 49]. Furthermore, the presence of a spiritual environment is reported to be associated with positive outcomes at both the individual and organizational levels. An environment where employees' spiritual needs are recognized and supported has been associated with meaningful work experiences, a sense of belonging, and quality of interaction; this, in turn, has been linked to job satisfaction, ethical sensitivity, and organizational citizenship behavior [50, 51]. However, environmental factors such as heavy workloads, long working hours, and frequent shifts have been negatively related to nurses' perceptions of spiritual care. Yıldırım and Ertem's study further revealed that such conditions are negatively related to spiritual sensitivity and professional satisfaction [49]. Therefore, it is clear that institutions need to strengthen psychosocial and spiritual support systems beyond physical and structural conditions.

In this context, the mediating role of spirituality can be explained based on several key functions. First, spirituality reconstructs the meaning of work, enabling nurses to view their daily tasks as more fulfilling and valuable services [51]. Spiritual practices (meditation, mindfulness, and prayer) have been found to be positively associated with individuals' stress management skills and mental and emotional strength, and they have also been linked to nurses' overall well-being and professional performance [52]. Third, the alignment between organizational culture and individual values has been reported to be related to employees' sense of community, which in turn is associated with professional attitudes such as organizational commitment, motivation, ethical sensitivity, and citizenship behavior, as well as work–life quality [53]. This suggests that work–life quality in nursing is shaped not only by structural and environmental factors but also by individual spiritual resources and the organizational spiritual climate, and that this multidimensional interaction is associated with sustainable well-being.

The Employee Spirituality Scale utilized in this study and the participants' replies warrant meticulous assessment. The statements in the “Relationship with Divine Power” subdimension of the scale may be theoretically construed in several manners contingent upon the participant's belief system (e.g., God, universal energy, the universe, inner self, etc.). These comments may predominantly be associated with religiosity in nations such as Türkiye, where religious themes permeate daily life [54]. For instance, statements such as “Divine Power influences my decisions at work” or “I seek assistance from Divine Power” may have been perceived by participants as indicative of religious belief rather than spiritual experience. Approximately half of the participants indicated active engagement in everyday spiritual practices, thus reinforcing this image. This situation aligns with existing studies indicating that the terms “spirituality” and “religion” are frequently utilized interchangeably within the Turkish context [54, 55]. Consequently, while the scale is ostensibly secular in nature (e.g., business ethics, social responsibility, and human connections), participants may have, in fact, viewed these items predominantly via a religious lens. This indicates that the understanding of spirituality is influenced by cultural context. In nations with pronounced religious influences, such as Turkey, spirituality predominantly aligns with religion [56]; conversely, in a Western setting, it is often linked to the pursuit of personal meaning or secular activities [57].

Participants in this study were recruited through social media. This strategy could have led to a bias in the sample, favoring nurses with greater digital engagement and frequent use of online platforms. The prevalence of young (mean age ≈ 29.5), mostly female (69.1%), nurses employed in public hospitals (68.9%) within the sample corroborates this selection impact. The results may represent the experiences of more digitally engaged demographics, concerning the interplay between spirituality, work environment, and quality of life.

### 4.1. Limitations

This study has some limitations. First, the data were collected using self-report scales. Given that participants may be inclined to provide socially desirable responses, this raises the risk of social desirability bias or respondent bias. Second, the study employed a cross-sectional research design. Therefore, the relationships between variables should be interpreted as correlational rather than causal. Longitudinal studies are needed in the future to test these relationships more robustly. Third, the study was conducted solely on a Turkish sample. When evaluated from the perspective of a culturally sensitive concept such as spirituality, the generalizability of the findings to different cultural contexts is limited. In this regard, comparative studies involving different countries and cultural groups in future research could offer valuable insights in terms of cross-cultural validity. Fourth, spirituality is an individual, multilayered, and subjective concept; thus, scales may be insufficient to fully capture its dimensions. Therefore, it is important to employ qualitative research approaches to explore the more subjective, in-depth, and multidimensional aspects of spirituality. In particular, phenomenological or narrative-based studies may provide a better understanding of nurses' personal spiritual experiences. Finally, the participants in this study were recruited via WhatsApp and social media. This sampling method may have introduced a self-selection bias, as participation was likely influenced by individuals' own willingness to engage. Consequently, individuals who use digital platforms less frequently may have been underrepresented, which could limit the generalizability of the findings.

### 4.2. Effects of Nursing Management

The results of this study reveal that improving the quality of working life requires addressing not only the physical but also the spiritual needs of nurses. In this regard, nurse managers must assume an active leadership role in creating supportive environments that respond to nurses' spiritual needs, in addition to enhancing their physical working conditions. Establishing spiritual support systems not only for patients but also for nurses is critically important for professional sustainability and emotional resilience.

In this context, it is essential to develop inclusive practices that accommodate various spiritual orientations. Structures such as spiritual counseling services, quiet rooms, meditation spaces, and stress management or mindfulness workshops can contribute to nurses' inner balance and resilience. Institutions that implement such practices also cultivate a meaningful and supportive corporate culture aligned with the values of their employees.

In organizations where a spiritual climate is embedded in the corporate culture, fundamental principles such as meaningfulness, empathy, justice, respect for diverse beliefs, and support for internal sources of strength become prominent. Such an environment enables nurses to draw more effectively on their internal resources, resulting in positive outcomes in job satisfaction, organizational commitment, and professional motivation. Ultimately, this increase in employee satisfaction will directly enhance the quality of patient care and contribute to the overall improvement of healthcare services.

## 5. Conclusions

This study has shed light on the multidimensional nature of nurses' professional experiences, the relationship between the nursing work environment and work–life quality, and the potential mediating role of spirituality in this relationship. The findings indicate that a positive work environment is significantly associated with nurses' work–life quality, both directly and indirectly through spirituality. Making clinical environments sensitive to spiritual needs can positively contribute to the overall quality of healthcare services by improving nurses' work–life quality.

In this context, it is important to develop inclusive practices that address diverse spiritual orientations and train leaders capable of fostering spiritually supportive work environments. Such practices will also lay the foundation for the development of a positive spiritual climate within institutions. Future research should investigate the role of managerial attitudes and institutional policies in shaping this climate. These studies will contribute to the development of a more holistic perspective on building a spiritual climate in healthcare institutions.

## Figures and Tables

**Table 1:** Characteristics of participants (*N* = 350).

	Statistics
Age, mean ± standard deviation (min–max)	29.53 ± 5.17 (22–38)
Gender, *n* (%)	
Female	242 (69.1%)
Male	108 (30.9%)
Marital status, *n* (%)	
Married	158 (45.1%)
Single	192 (54.9%)
Educational level, *n* (%)	
Secondary education	10 (2.9%)
Undergraduate	306 (43.2%)
Postgraduate	34 (9.7%)
Income level, *n* (%)	
Income less than expenses	94 (26.9%)
Income equal to expenses	221 (63.1%)
Income more than expenses	35 (10.0%)
Professional experience (years), *n* (%)	
Less than 1 year	34 (9.7%)
1–5 years	104 (29.7%)
6–10 years	186 (53.1%)
11 years and above	26 (7.4%)
Type of institution, *n* (%)	
Public hospital	241 (68.9%)
Private hospital	49 (14.0%)
University hospital	60 (17.1%)
Unit of work, *n* (%)	
Intensive care unit	49 (14.0%)
Emergency department	35 (10.0%)
Internal medicine clinic	130 (37.1%)
Surgical clinic	116 (33.1%)
Operating room	20 (5.7%)
Weekly working hours, *n* (%)	
40 h	45 (12.9%)
41–50 h	147 (42.0%)
51–60 h	96 (27.4%)
61 h and above	62 (17.7%)
Perceived importance of spirituality, *n* (%)	
Yes, important	234 (66.9%)
No, not important	39 (11.1%)
Undecided	77 (22.0%)
Frequency of spiritual practices, *n* (%)	
Daily	172 (49.1%)
Weekly	60 (17.1%)
Monthly	46 (13.1%)
Rarely	34 (9.7%)
Never	38 (10.9%)

**Table 2:** Correlations among variables (*N* = 350).

		Work environment						Quality of working life						Spirituality		
		1	2	3	4	5	6	7	8	9	10	11	12	13	14	15
Work environment	1	1														
	2	0.79^∗^	1													
	3	0.85^∗^	0.85^∗^	1												
	4	0.84^∗^	0.85^∗^	0.92^∗^	1											
	5	0.81^∗^	0.80^∗^	0.89^∗^	0.89^∗^	1										
	6	0.93^∗^	0.94^∗^	0.95^∗^	0.94^∗^	0.91^∗^	1									

Quality of working life	7	0.24^∗^	0.21^∗^	0.26^∗^	0.26^∗^	0.22^∗^	0.25^∗^	1								
	8	0.24^∗^	0.21^∗^	0.21^∗^	0.20^∗^	0.16^∗∗^	0.20^∗^	0.43^∗^	1							
	9	0.24^∗^	0.22^∗^	0.26^∗^	0.27^∗^	0.24^∗^	0.26^∗^	0.44^∗^	0.39^∗^	1						
	10	0.20^∗^	0.17^∗∗^	0.20^∗^	0.22^∗^	0.20^∗^	0.21^∗^	0.39	0.35^∗^	0.40^∗^	1					
	11	0.20^∗^	0.17^∗∗^	0.22^∗^	0.22^∗^	0.21^∗^	0.21^∗^	0.50^∗^	0.47^∗^	0.41^∗^	0.464^∗^	1				
	12	0.30^∗^	0.27^∗^	0.32^∗^	0.32^∗^	0.29^∗^	0.31^∗^	0.81^∗^	0.64^∗^	0.78^∗^	0.693^∗^	0.70^∗^	1			

Spirituality	13	0.45^∗^	0.42^∗^	0.46^∗^	0.45^∗^	0.44^∗^	0.47^∗^	0.18^∗∗^	0.12^∗∗∗^	0.28^∗^	0.172^∗∗^	0.17^∗∗^	0.25^∗^	1		
	14	0.55^∗^	0.51^∗^	0.59^∗^	0.59^∗^	0.57^∗^	0.59^∗^	0.26^∗^	0.17^∗∗^	0.26^∗^	0.239^∗^	0.19^∗^	0.32^∗^	0.70^∗^	1	
	15	0.54^∗^	0.50^∗^	0.57^∗^	0.56^∗^	0.54^∗^	0.57^∗^	0.23^∗^	0.15^∗∗^	0.26^∗^	0.219	0.19^∗^	0.30^∗^	0.94^∗^	0.91^∗^	1

*Note,* 1, Participation in governance and perceived influence; 2, nursing resources for quality care; 3, leadership approaches of nurse managers; 4, adequacy of staffing and resources; 5, physician–nurse–colleague communication; 6, Nursing Work Environment Assessment Scale; 7, work/workplace environment; 8, relationships with managers; 9, working conditions; 10, job perception; 11, support services; 12, Nursing Work–Life Quality Scale; 13, relationship with divine power; 14, attitudes toward colleagues and employer; 15, Employee Spirituality Scale.

^∗^ *p* < 0.001.

^∗∗^ *p* < 0.01.

^∗∗∗^ *p* < 0.05.

**Table 3:** Goodness-of-fit statistics for the structural equation model (*N* = 350).

Indices	*χ* ^2^	df	*p*-value	*χ* ^2^/df	RMSEA	SRMR	CFI	TLI	NFI	GFI	AGFI
Values of the model	52.78	51	0.405	1.034	0.010	0.023	0.976	0.980	0.963	0.971	0.961
Acceptable values	—	—	> 0.05	< 3	< 0.08	< 0.08	> 0.90	> 0.90	> 0.90	> 0.90	> 0.90

**Table 4:** Path analysis.

Path	Effect (unstandardized *β*)	SE	CR	*p*	Effect (standardized *β*)
Work environment ⟶ spirituality	0.65	0.037	17.528	< 0.001	0.648
Work environment ⟶ quality of working life	0.2	0.089	2.467	0.014	0.22
Spirituality ⟶ quality of working life	0.198	0.093	2.309	0.021	0.216

**Table 5:** The mediating effect of spirituality.

Path/effect	Effect coefficient (*β*)	Standard error (SE)	*p*	95% Confidence interval
Direct effects				
Work environment ⟶ quality of working life	0.220	0.089	0.014	[0.045, 0.394]
Work environment ⟶ spirituality	0.648	0.037	< 0.001	[0.575, 0.720]
Spirituality ⟶ quality of working life	0.216	0.093	0.021	[0.033, 0.399]
Indirect effect				
Work environment ⟶ spirituality ⟶ quality of working life	0.201	0.092	0.029	[0.024, 0.390]
Total effect				
Work environment ⟶ quality of working life	0.516	0.089	< 0.001	[0.348, 0.703]

## Data Availability

The data that support the findings of this study are available from the corresponding author upon reasonable request.
